# Cardiovascular Dysfunction in Polycystic Ovary Syndrome: Mitochondrial and Inflammatory Mechanisms

**DOI:** 10.1155/bmri/1110229

**Published:** 2026-01-26

**Authors:** Olabimpe Caroline Badejogbin, Mary Olaoluwa Agunloye, Ojichukwuka Ebere Chijioke-Agu, Makinde Vincent Olubiyi, Success Oluwanifesimi Olugbuyiro, Olaniyi Azeez Soetan, Opeyemi Abel Bamgbose, Tobi Opeyemi Olaleye

**Affiliations:** ^1^ Department of Physiology, School of Basic Medical Sciences, Babcock University, Ilishan-Remo, Ogun State, Nigeria, babcock.edu.ng; ^2^ Department of Physiology, Kampala International University, Western Campus, Ishaka, Uganda, kiu.ac.ug; ^3^ Department of Physiology, Alex Ekwueme Federal University, Ebonyi State, Nigeria; ^4^ Department of Medical Physiology, University of Rwanda, Huye, Rwanda, ur.ac.rw; ^5^ Department of Physiology, College of Medicine, Olabisi Onabanjo University, Ago-Iwoye, Ogun State, Nigeria, oouagoiwoye.edu.ng; ^6^ Department of Physiology, Faculty of Basic Medical Sciences, College of Medicine, Achievers University, Owo, Ondo State, Nigeria, medcol.mw

**Keywords:** cardiovascular dysfunction, chronic inflammation, insulin resistance, mitochondrial dysfunction, oxidative stress, polycystic ovary syndrome, therapeutic targets

## Abstract

**Background:**

Polycystic ovary syndrome (PCOS) is a common endocrine disorder that significantly increases cardiovascular disease (CVD) risk in women. While insulin resistance and dyslipidemia are established contributors, growing evidence highlights mitochondrial dysfunction and chronic low‐grade inflammation as central drivers of cardiovascular pathology in PCOS.

**Objective:**

This narrative review synthesizes current evidence on how mitochondrial dysfunction and inflammation interact to promote cardiovascular complications in women with PCOS while identifying potential therapeutic targets and areas requiring further investigation.

**Methods:**

A comprehensive review of clinical and experimental studies was conducted using PubMed, Scopus, and Web of Science databases. Relevant literature exploring mitochondrial alterations, oxidative stress, inflammatory cytokines, and endothelial function in PCOS, with emphasis on cardiovascular outcomes, was critically evaluated and summarized.

**Results:**

Women with PCOS exhibit altered mitochondrial dynamics, reduced ATP production, and elevated reactive oxygen species (ROS), which collectively impair vascular function. These mitochondrial abnormalities compromise oocyte quality and endometrial receptivity and activate proinflammatory signaling pathways, including the NLRP3 inflammasome, contributing to endothelial dysfunction and atherogenesis, and increased long‐term cardiovascular risk, particularly in women with prior pregnancy complications. Elevated levels of cytokines including TNF‐*α*, IL‐6, and CRP further exacerbate cardiovascular risk. This bidirectional relationship between mitochondrial dysfunction and inflammation establishes a vicious cycle underlying cardiovascular deterioration in PCOS.

**Conclusion:**

Mitochondrial dysfunction and inflammation are interdependent mechanisms that contribute substantially to cardiovascular risk in women with PCOS. Targeting mitochondrial dysfunction and systemic inflammation presents a promising therapeutic strategy for reducing cardiovascular morbidity in PCOS. Future research should emphasize phenotype‐specific interventions, biomarker discovery, and translational trials to improve long‐term reproductive and cardiovascular outcomes.

## 1. Introduction

Polycystic ovary syndrome (PCOS) is a complex reproductive disorder affecting 6%–21% of women of reproductive age. It is marked by a combination of endocrine and metabolic abnormalities, including hyperandrogenism, polycystic ovaries, anovulation or oligomenorrhea, insulin resistance (IR), obesity, hirsutism, acne, and mood disturbances [1–3]. Women with hirsutism and irregular cycles often exhibit thecal and stromal cell hyperplasia, elevated testosterone and luteinizing hormone (LH), and reduced estradiol and FSH levels [4]. Disruption of the hypothalamic–pituitary–gonadal (HPG) axis impairs gonadotropin‐releasing hormone (GnRH) and gonadotropin secretion, contributing to anovulation and amenorrhea [5, 6]. These hormonal imbalances hinder follicular development, leading to cyst formation, menstrual irregularities, subfertility, increased risks of pregnancy complications such as miscarriage, and gestational diabetes [7, 8].

Beyond reproductive irregularities, infertility remains one of the most distressing outcomes of PCOS with hyperandrogenism and oxidative stress impairing oocyte quality and endometrial receptivity, resulting in poor fertilization, implantation, and pregnancy outcomes [9–11]. Mitochondrial dysfunction in granulosa and cumulus cells disrupts ATP production and calcium homeostasis, crucial for oocyte maturation, while oxidative damage compromises mitochondrial DNA (mtDNA) integrity and spindle organization, contributing to reduced oocyte competence and early pregnancy loss [10, 11]. In addition, women with PCOS frequently experience metabolic and vascular complications during pregnancy, including preeclampsia and preterm delivery, reflecting systemic IR and endothelial dysfunction [12]. Recent longitudinal studies further indicate that pregnancy may act as a cardiometabolic stress test, unmasking endothelial dysfunction and IR, thereby increasing the long‐term risk of Type 2 diabetes, hypertension, and cardiovascular disease (CVD) in women with PCOS [13, 14].

The impact of PCOS extends across a woman′s lifespan, predisposing affected individuals to serious health conditions, including cardiovascular and metabolic disorders such as metabolic syndrome, obesity, Type 2 diabetes, cardiovascular risks, depression, obstructive sleep apnea, endometrial cancer, and metabolic dysfunction–associated steatotic liver disease [15, 16]. Chronic low‐grade inflammation, oxidative stress, and endothelial dysfunction represent key mechanisms linking PCOS to these comorbidities, promoting hypertension, IR, and atherosclerosis, major contributors to CVD [17, 18]. The hyperandrogenic phenotype, in particular, is closely associated with cardiometabolic disturbances such as dyslipidemia, elevated blood pressure, and impaired glucose tolerance. Importantly, elevated cardiovascular risk, including coronary artery disease and stroke, persists in women with PCOS even after adjusting for conventional risk factors like BMI and is evident across all phenotypes [19, 20].

CVD remains the leading global cause of death, driving significant health loss and economic burden [21]. Heart failure (HF), a major form of CVD, affects over 64 million adults worldwide and often coexists with chronic kidney disease (CKD), which is present in about 50% of HF patients and markedly increases mortality and cardiovascular risk [22, 23]. The interplay between cardiac and renal dysfunction, termed cardiorenal syndrome, reflects a bidirectional relationship where dysfunction in one organ exacerbates the other [24]. While HF has long been recognized as a trigger for kidney damage, current understanding acknowledges that both the heart and kidneys can initiate this pathological cycle. CKD significantly heightens CVD risk, with nearly half of CKD‐related deaths attributed to major cardiovascular events, largely driven by aging and metabolic disorders [23]. Chronic inflammation plays a central role in the progression of both HF and CKD, promoting tissue injury, fibrosis, and organ failure through sustained release of proinflammatory mediators [25, 26].

In PCOS, disease progression and systemic complications are driven by a complex interplay of IR, hyperandrogenism, chronic inflammation, and dysfunctional adipose tissue [27]. Oxidative stress, primarily generated through mitochondrial dysfunction, serves as a central mechanism linking metabolic, reproductive, and cardiovascular abnormalities in PCOS. While reactive oxygen species (ROS) play physiological roles in gene expression, oocyte maturation, and cellular maturation, excessive ROS disrupts cellular homeostasis, induces apoptosis, and activates proinflammatory cytokines such as tumor necrosis factor‐*α* (TNF‐*α*), contributing to both ovarian and cardiovascular dysfunction [28–30]. In granulosa cells of PCOS ovaries, elevated ROS impairs HIF‐1*α*‐mediated mitophagy, leading to the accumulation of damaged mitochondria and further oxidative injury, which promotes ovarian failure [31, 32]. These mitochondrial defects, reflected by downregulation of fusion proteins like MFN2, further exacerbate IR and endocrine disruption [33, 34]. As a result, disrupted mitochondrial quality control fosters sustained ROS production, impaired follicular development, and cyst formation, leading to ovarian insufficiency characterized by elevated anti‐Müllerian hormone (AMH) and hypoestrogenemia [35].

Cardiovascular abnormalities in PCOS are also evident in structural and functional markers. Women with PCOS exhibit increased coronary artery calcification and carotid intima–media thickness (CIMT), both early indicators of atherosclerosis and elevated CVD risk [36–38]. Endothelial dysfunction, a hallmark of early CVD, manifests as reduced nitric oxide (NO) bioavailability and lower endothelial nitric oxide synthase (eNOS) expression, often accompanied by increased cardiac troponin T concentrations [39, 40]. These changes are consistent with hyperinsulinemia‐ and IR‐mediated cardiac dysfunction [41, 42], highlighting the progressive nature of cardiovascular complications in PCOS [43].

Thus, the interplay between mitochondrial dysfunction, oxidative stress, chronic inflammation, and hyperandrogenism constitutes a unifying mechanism underlying both reproductive and cardiovascular abnormalities in PCOS. Understanding these interconnections may facilitate early risk stratification and guide development of targeted interventions, particularly for women with hyperandrogenic phenotypes or prior pregnancy complications. This review synthesizes current clinical and experimental evidence linking mitochondrial and inflammatory mechanisms to cardiovascular dysfunction in PCOS, emphasizing their pathophysiological relevance, molecular interactions, and therapeutic implications.

## 2. Methodology

This work was conducted as a narrative (nonsystematic) review, aimed at synthesizing current evidence on cardiovascular dysfunction in PCOS, particularly in relation to mitochondrial and inflammatory mechanisms. Because the available literature is heterogeneous and spans experimental, clinical, and translational studies, a narrative approach was chosen to allow flexible expert‐driven selection and integrative interpretation of relevant findings.

To ensure breadth of coverage, we conducted a broad, nonsystematic search of the literature using electronic databases such as PubMed, Scopus, Web of Science, and Google Scholar [44–48]. Search terms included combinations of “polycystic ovary syndrome,” “PCOS,” “cardiovascular disease,” “oxidative stress,” “mitochondrial dysfunction,” “chronic inflammation,” and “endothelial dysfunction.” The search was not restricted by a predefined protocol, and inclusion was based on the authors′ judgment of relevance to PCOS‐related cardiovascular and metabolic dysfunction. Studies were considered if they provided mechanistic, clinical, or pathophysiological insights into oxidative stress, inflammation, mitochondrial abnormalities, or endothelial dysfunction in PCOS. Articles unrelated to PCOS biology, male‐only studies, or evidence lacking relevance to cardiometabolic pathways were excluded at the authors′ discretion. This selective process was not systematic, did not follow PRISMA guidelines, and was not registered in PROSPERO.

**Figure 1:**
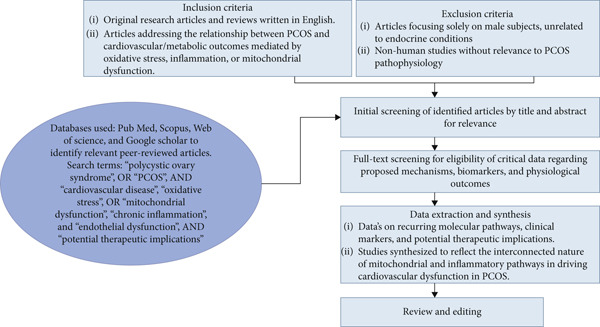
Overview of the nonsystematic literature selection process used in this narrative review.

Titles and abstracts were reviewed to identify studies of interest, followed by full‐text reading. Key findings were synthesized thematically, allowing the integration of molecular, clinical, and mechanistic data. A simplified overview of this nonsystematic literature selection process is illustrated in Figure 1, which visually summarizes the narrative steps used to identify and select relevant studies based on expert judgment. This narrative synthesis approach enabled the identification of recurrent pathways and emerging therapeutic implications linking mitochondrial dysfunction, chronic inflammation, and cardiovascular risk in women with PCOS.

## 3. Endocrine Dysregulation and Cardiometabolic Consequences in PCOS

Hyperinsulinemia is a central driver of the endocrine and metabolic dysregulation observed in PCOS, primarily through disruption of the HPG axis and consequent reproductive impairment [6]. In PCOS, IR induces compensatory hyperinsulinemia, which sensitizes ovarian theca cells to LH, amplifying androgen synthesis and exacerbating hyperandrogenic symptoms [49, 50]. Insulin acts synergistically with LH to stimulate androgen production and modulates GnRH pulsatility, resulting in an increased LH‐to‐FSH ratio. Additionally, insulin enhances adrenal responsiveness to adrenocorticotropic hormone (ACTH), further promoting androgen excess.

Hyperinsulinemia suppresses hepatic production of sex hormone–binding globulin (SHBG) via downregulation of hepatocyte nuclear factor 4‐alpha (HNF‐4*α*), increasing circulating free testosterone. Reduced insulin‐like growth factor–binding Protein 1 (IGFBP‐1) simultaneously augments IGF‐1 activity, stimulating ovarian steroidogenesis and contributing to hyperandrogenemia [51]. Insulin also upregulates key steroidogenic genes in the ovarian theca cells, further reinforcing androgen excess. Elevated testosterone and diminished SHBG, hallmarks of this hyperinsulinemic state, impair follicular development and ovulatory function [6, 52, 53].

Beyond reproductive consequences, IR precipitates metabolic dysfunction, predisposing women with PCOS to Type 2 diabetes, dyslipidemia, hypertension, and atherosclerosis [54, 55]. Altered adipocyte metabolism enhances lipolysis, elevates circulating triglycerides and low‐density lipoprotein cholesterol (LDL‐C), and reduces high‐density lipoprotein cholesterol (HDL‐C), fostering an atherogenic lipid profile [23, 55–58]. This dyslipidemia promotes oxidative stress through ROS accumulation, depleting antioxidant reserves and initiating endothelial dysfunction [59–61]. Globally, atherogenic dyslipidemia accounts for a significant proportion of ischemic heart disease and stroke, highlighting its relevance to cardiovascular risk in PCOS [62–64].

Hyperandrogenism, present in 60%–80% of women with PCOS, further amplifies metabolic and reproductive abnormalities. Theca cells′ heightened responsiveness to insulin and gonadotropins leads to ovarian hyperthecosis and excessive androgen production [6]. Clinically, hyperandrogenism manifests as hirsutism, acne, menstrual irregularities, and anovulation [65]. Elevated testosterone correlates with increased antral follicle counts and disrupted granulosa cell function, impairing follicular maturation and promoting follicular atresia [66, 67]. Excess estrogen from these follicles suppresses FSH through negative feedback, perpetuating ovulatory dysfunction. Polycystic ovaries are defined by ≥ 12 antral follicles (2–9 mm) and/or ovarian volume > 10 mL, key diagnostic criteria [68]. IR and hyperandrogenism synergistically exacerbate cardiometabolic risk. Hyperinsulinemia promotes hepatic VLDL synthesis, increasing triglycerides and LDL‐C while lowering HDL‐C, contributing to atherogenic dyslipidemia [69]. It also drives hypertension via renal sodium retention, impaired NO bioavailability, and autonomic dysregulation [69]. Androgen excess further modifies lipid metabolism and insulin sensitivity through androgen receptor–mediated mechanisms [70].

**Figure 2:**
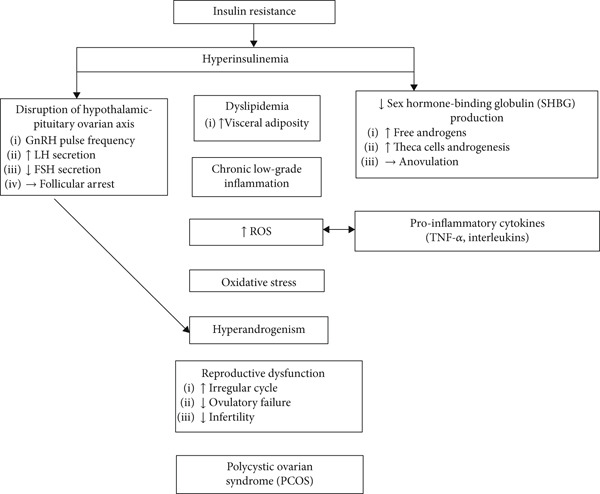
Overview of pathophysiological mechanisms in PCOS.

Emerging evidence highlights subclinical cardiovascular abnormalities in women with PCOS, including increased CIMT and elevated inflammatory and endothelial injury biomarkers, such as endothelin‐1 and soluble lectin–like oxidized LDL receptor‐1 (sLOX‐1), reflecting early vascular dysfunction [15, 71–75]. Longitudinal studies confirm heightened incidence of coronary heart disease and stroke in PCOS, independent of BMI, emphasizing the roles of IR, hyperandrogenism, and chronic low‐grade inflammation as central mediators of cardiovascular risk [42, 76–81]. In summary, the endocrine profile of PCOS represents a pathological triad of hyperinsulinemia, IR, and hyperandrogenism. These interconnected mechanisms disrupt reproductive function, impair folliculogenesis, and promote visceral adiposity, dyslipidemia, and endothelial dysfunction, cumulatively elevating cardiometabolic risk. Figure 2 illustrates how hyperinsulinemia and IR converge with hyperandrogenism, oxidative stress, and proinflammatory cytokines to impair reproductive function and potentiate cardiovascular complications.

## 4. Oxidative Stress and Mitochondrial Dysfunction in PCOS

Oxidative stress represents a central mechanistic link between PCOS and CVD. In women with PCOS, excessive production of ROS compromises cellular and vascular integrity, promoting lipid peroxidation, protein modification, and DNA damage [82]. Mitochondria, as the primary sites of ATP synthesis, are both a major source and a target of ROS. In PCOS, mitochondrial dysfunction exacerbates ROS accumulation, impairing oxidative phosphorylation, reducing ATP output, and initiating a self‐perpetuating cycle of oxidative injury [83]. Normally, ROS are produced at Complexes I and III of the electron transport chain (ETC) [84], but in PCOS, defective mitochondrial respiration increases ROS generation, promoting cardiomyocyte and endothelial injury. This contributes to myocardial hypertrophy, cardiac fibrosis, and vascular remodeling, hallmarks of early cardiovascular dysfunction [85].

Clinical studies demonstrate elevated oxidative biomarkers in PCOS, including malondialdehyde (MDA), with concomitant reductions in antioxidant defenses such as superoxide dismutase (SOD) and glutathione (GSH) [86]. This imbalance facilitates LDL oxidation, foam cell formation, and vascular inflammation, accelerating atherogenesis [82]. Oxidative stress also impairs cardiomyocyte calcium handling and mitochondrial function, further promoting cardiac fibrosis and hypertrophy [85].

The generation of ROS in PCOS stems from the intersection of IR, hyperandrogenism, and chronic low‐grade inflammation. Hyperinsulinemia and inflammatory cytokines upregulate NADPH oxidase (NOX) activity, producing ROS independently of mitochondria [87, 88]. Hyperglycemia further promotes advanced glycation end‐product (AGE) formation, which binds to RAGE on endothelial cells, activating NF‐*κ*B and amplifying proinflammatory gene expression [89]. These pathways converge to accelerate endothelial dysfunction, linking oxidative stress with cardiovascular risk in PCOS.

IR also impairs ETC efficiency, causing electron leakage and ROS accumulation [90]. Hyperandrogenism exacerbates these effects by increasing visceral adiposity and systemic inflammation, inhibiting mitochondrial Complex I, reducing ATP production, and downregulating antioxidant enzymes [91]. The coexistence of hyperandrogenism and IR amplifies mitochondrial ROS generation, creating a feedback loop that sustains oxidative stress and metabolic dysfunction [85, 89].

**Figure 3:**
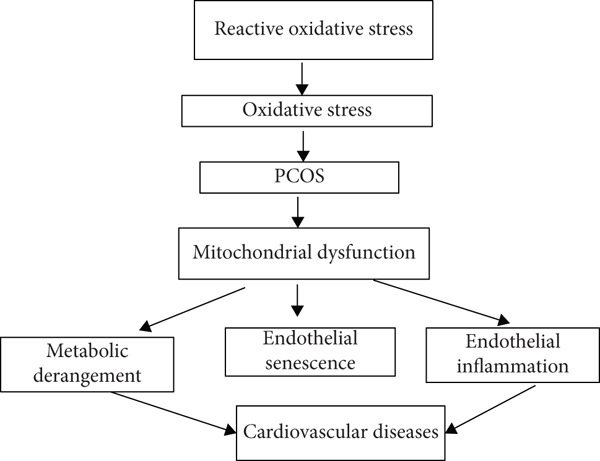
Oxidative stress and mitochondrial dysfunction as mediators of cardiovascular risk in PCOS.

Figure 3 shows how oxidative stress, mitochondrial dysfunction, IR, and hyperandrogenism interconnect to elevate cardiovascular risk.

## 5. Chronic Inflammation–Mitochondrial Dysfunction Axis and Cardiovascular Complications in PCOS

PCOS is increasingly recognized as a systemic cardiometabolic disorder in which chronic inflammation and mitochondrial dysfunction intersect to drive vascular injury, endothelial dysfunction, and increased long‐term CVD risk. The persistence of low‐grade inflammation, combined with metabolic and endocrine abnormalities, establishes a pathogenic environment favoring oxidative stress, mitochondrial impairment, and progressive vascular remodeling [92, 93].

Women with PCOS exhibit elevated circulating inflammatory biomarkers, including C‐reactive protein (CRP), interleukin‐6 (IL‐6), interleukin‐18 (IL‐18), and TNF‐*α*, reflecting the chronic activation of immune and metabolic pathways [94, 95]. These cytokines promote serine phosphorylation of insulin receptor substrate‐1 (IRS‐1), impair phosphatidylinositol 3‐kinase (PI3K) signaling, and aggravate IR, forming a feedback loop in which inflammation amplifies metabolic dysfunction [96, 97].

Atherosclerotic processes are initiated by subendothelial retention of LDL particles, which undergo oxidative modification and trigger endothelial activation [98]. Oxidized LDL promotes leukocyte recruitment, macrophage activation, and foam cell formation, facilitating plaque expansion. The formation of cholesterol crystals activates the NLRP3 inflammasome, generating interleukin‐1*β* (IL‐1*β*) and IL‐18, which further intensify arterial inflammation [99, 100].

Immune dysregulation contributes to this proinflammatory state. Women with PCOS demonstrate increased M1 macrophage polarization, heightened Th1 and Th17 responses, reduced Treg activity, and enhanced B‐cell activation, all of which promote endothelial injury and atherosclerotic risk [96, 101, 102]. Neutrophils contribute to vascular damage through degranulation, production of ROS, and formation of neutrophil extracellular traps (NETs), while dendritic cells perpetuate proinflammatory signaling through antigen presentation and cytokine release [103, 104].

Central to this inflammatory environment is mitochondrial dysfunction, which functions both as a cause and a consequence of oxidative stress and immune activation. Mitochondria play critical roles in oxidative phosphorylation, redox regulation, calcium handling, apoptosis, and innate immune sensing [105]. In PCOS, impaired ETC activity, particularly at Complexes I and III, leads to excessive ROS production, reduced ATP synthesis, and accumulation of mitochondrial damage [106]. Excess mitochondrial ROS react with NO to form peroxynitrite (ONOO^-^), a potent oxidant that diminishes NO bioavailability, disrupts vasodilation, and promotes endothelial dysfunction and arterial stiffness [106, 107]. Damaged mitochondria release mtDNA and cardiolipin, which act as damage‐associated molecular patterns (DAMPs) that activate Toll‐like receptors (TLRs), NLRP3 inflammasomes, and the cGAS–STING pathway, propagating systemic inflammation and vascular injury [108]. Mitochondrial quality control mechanisms, including biogenesis, fission, fusion, and mitophagy, are impaired in PCOS. Dysfunctional mitophagy allows accumulation of fragmented, depolarized mitochondria that further enhance ROS generation and endothelial injury [109]. Persistent oxidative stress leads to lipid peroxidation, protein carbonylation, and mtDNA mutations, perpetuating a vicious cycle of mitochondrial deterioration and chronic inflammation [110]. These mitochondrial disturbances extend to ovarian granulosa cells, where disrupted mitochondrial morphology, impaired OXPHOS, and insufficient mitophagy reduce oocyte competence and follicular maturation, contributing to reproductive dysfunction [111].

Hyperandrogenism intensifies mitochondrial dysfunction by increasing visceral adiposity, inhibiting mitochondrial Complex I, elevating ROS production, and reducing antioxidant enzyme activity (SOD, GPx, and catalase), linking endocrine abnormalities to cardiovascular and metabolic pathology [91]. Cardiac tissue is similarly affected. Reduced ATP synthesis, elevated mitochondrial ROS, impaired mitochondrial membrane potential, and increased susceptibility to apoptosis compromise cardiomyocyte function, predisposing women with PCOS to hypertension, ventricular hypertrophy, ischemic heart disease, and HF [112]. During ischemia–reperfusion injury, compromised mitochondrial resilience results in more severe oxidative damage compared with non‐PCOS controls [113]. Mitochondrial dysfunction also drives immune‐metabolic reprogramming, shifting macrophages toward glycolytic metabolism, which supports increased proinflammatory cytokine production and reinforces systemic inflammation [114]. This establishes a feedforward loop in which metabolic dysfunction, immune activation, and mitochondrial injury perpetuate one another.

Recent evidence identifies chemerin as a novel adipokine with significant relevance in PCOS. Chemerin regulates adipogenesis, glucose metabolism, and immune cell chemotaxis. Elevated chemerin levels in PCOS correlate with IR, inflammation, and endothelial dysfunction and may serve as a biomarker for cardiovascular risk [115]. Its receptors, chemokine‐like Receptor 1 (CMKLR1) and chemokine receptor‐like 2 (CCRL2), are expressed in endothelial and ovarian tissue, mediating adipose inflammation and vascular remodeling. Chemerin thus represents an emerging therapeutic and diagnostic target in PCOS‐associated cardiometabolic dysfunction [115]. Therapeutically, GLP‐1 receptor agonists (liraglutide and semaglutide) have demonstrated benefits in weight reduction, insulin sensitivity, inflammation, and endothelial function [116]. Although most trials were conducted in general obese or diabetic populations, not exclusively in PCOS women, clinical evidence suggests that GLP‐1 agonists reduce visceral adiposity, lower inflammatory cytokines, enhance mitochondrial biogenesis, and improve lipid profiles, mechanisms directly relevant to PCOS pathophysiology [117]. Their expanding use in PCOS requires clarification when evidence derives from non‐PCOS cohorts.

Mitochondria‐targeted antioxidants such as MitoQ and elamipretide (SS‐31) reduce oxidative stress, preserve membrane potential, and have shown therapeutic potential in restoring mitochondrial function [118]. Agents activating peroxisome proliferator–activated receptor gamma coactivator 1‐alpha (PGC‐1*α*) improve mitochondrial biogenesis, enhance oxidative phosphorylation efficiency, and reduce proinflammatory signaling [119]. Anti‐inflammatory biologics targeting IL‐1*β* protect mitochondrial structure from cytokine‐induced injury and may reduce vascular risk [120]. Lifestyle interventions, including regular exercise, caloric restriction, and weight loss, consistently enhance mitochondrial function, reduce inflammatory biomarkers, and improve cardiometabolic outcomes in PCOS [121].

**Figure 4:**
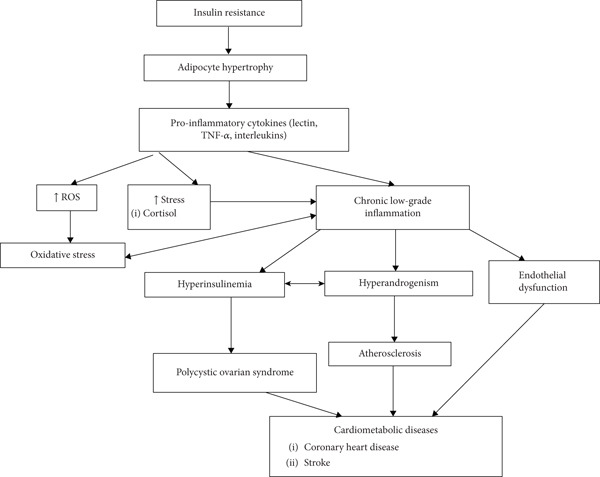
Pathogenesis of inflammation in PCOS‐associated cardiovascular disease.

The convergence of chronic inflammation, oxidative stress, and mitochondrial dysfunction is strongly associated with increased CIMT, endothelial dysfunction, arterial stiffness, early atherosclerosis, hypertension, and a heightened lifetime risk of coronary artery disease and stroke in women with PCOS [122, 123]. Figure 4 depicts the integrative pathway showing how IR, adipocyte hypertrophy, hyperandrogenism, mitochondrial dysfunction, oxidative stress, and chronic inflammation collectively drive cardiovascular complications in PCOS.

## 6. Therapeutic Approaches Targeting Mitochondrial and Inflammatory Pathways

### 6.1. Lifestyle Interventions: Diet, Exercise, and Weight Management

Lifestyle interventions are considered a primary approach in the therapeutic management of PCOS, owing to their broad positive effects on metabolic, cardiovascular, and reproductive health [124]. Since mitochondrial dysfunction and chronic inflammation are closely linked in the pathophysiology of PCOS, therapeutic strategies that improve cellular energy homeostasis and alleviate oxidative and inflammatory stress hold considerable potential for reducing cardiovascular complications [125].

#### 6.1.1. Diet and Nutritional Regulation

Nutritional habits play a critical role in regulating mitochondrial function and inflammatory processes [126, 127]. Diets high in calories and glycemic load can lead to mitochondrial stress, increased production of ROS, and lipid buildup, which collectively worsen IR and impair endothelial function [127]. Conversely, calorie‐controlled diets with a low glycemic index enhance mitochondrial efficiency and suppress the production of proinflammatory cytokines [128]. The Mediterranean diet, characterized by high intake of monounsaturated fats, polyphenols, and omega‐3 fatty acids, along with low consumption of red meat, has been associated with notable antioxidant and anti‐inflammatory properties [129]. This dietary pattern has been shown to improve lipid metabolism, vascular health, and insulin sensitivity in individuals with PCOS. Likewise, although still debated, the ketogenic diet has demonstrated temporary benefits in reducing visceral fat, oxidative stress, and inflammatory mediators, thereby indirectly supporting mitochondrial function and cardiovascular health [130–133]. Additionally, dietary supplementation with bioactive compounds such as omega‐3 fatty acids, coenzyme Q10 (CoQ10), and resveratrol, commonly found in functional foods, further enhances mitochondrial bioenergetics and inhibits key inflammatory pathways, including NF‐*κ*B and the NLRP3 inflammasome [134–140].

#### 6.1.2. Exercise and Mitochondrial Biogenesis

Engagement in both aerobic and resistance exercise stimulates mitochondrial biogenesis by activating PGC‐1*α*, a key transcriptional regulator of mitochondrial metabolism, and by upregulating endogenous antioxidant enzymes [141]. Regular physical activity suppresses proinflammatory adipokines such as TNF‐*α* and IL‐6, while enhancing the expression of anti‐inflammatory mediators like IL‐10 [142, 143]. In women with PCOS, consistent participation in moderate–intensity aerobic exercise has been associated with improvements in maximal oxygen uptake (VO_2_ max), reductions in central adiposity, enhanced insulin sensitivity, and normalization of lipid profiles [144–146]. Incorporating resistance training further augments mitochondrial density in skeletal muscle and facilitates glucose uptake, which are essential adaptations to counteract the metabolic inflexibility characteristic of PCOS [147]. Crucially, combined lifestyle strategies involving both structured exercise and dietary modifications exert synergistic benefits by promoting mitochondrial health, attenuating systemic inflammation, and enhancing cardiovascular function more effectively than either intervention alone [148, 149].

#### 6.1.3. Weight Management and Cardiovascular Restoration

Excess body weight (obesity), particularly visceral fat, plays a critical role in the disruption of mitochondrial homeostasis and the amplification of chronic inflammation in PCOS. Achieving even a modest reduction in body weight, typically between 5%, has been shown to significantly enhance endothelial function, lower circulating levels of ROS, and suppress the expression of inflammatory cytokines [150].

Weight loss contributes to improved vascular function through enhanced NO bioavailability, attenuation of autonomic nervous system imbalance, and reduction in arterial stiffness, all of which are key determinants of cardiovascular health in women with PCOS [151]. Furthermore, effective weight management has been shown to enhance the therapeutic efficacy of pharmacological treatments and positively influence reproductive outcomes [152].

### 6.2. Pharmacological Treatments: Metformin, Anti‐Inflammatory Agents, and Antioxidants

Addressing mitochondrial impairment and chronic inflammation through pharmacological interventions has become a promising strategy for reducing cardiovascular complications associated with PCOS. A range of therapeutic agents has shown effectiveness in regulating oxidative stress, restoring mitochondrial energy function, and alleviating systemic inflammation.

#### 6.2.1. Metformin: Extending Beyond Glycemic Regulation

Metformin continues to be the primary pharmacologic agent used in PCOS due to its broad spectrum of therapeutic actions. It functions by activating AMP‐activated protein kinase (AMPK), which enhances mitochondrial oxidative capacity, suppresses hepatic glucose production, and improves glucose uptake in peripheral tissues [153]. AMPK activation also stimulates mitochondrial biogenesis and autophagy, resulting in decreased production of mitochondrial reactive oxygen species (mtROS) and improved cellular redox homeostasis [153].

In the cardiovascular system, metformin has been shown to enhance eNOS activity, reduce vascular inflammation, and prevent endothelial cell apoptosis [154, 155]. Clinical trials have linked metformin use in PCOS patients with reductions in CIMT, arterial stiffness, and circulating inflammatory biomarkers such as CRP and TNF‐*α* [155, 156]. In addition, metformin modulates inflammatory pathways by inhibiting NF‐*κ*B signaling and mitigating vascular damage caused by AGEs, thereby contributing to improved vascular elasticity and a reduced long‐term cardiovascular risk [155, 156]. Importantly, recent evidence indicates that metformin administration before and during pregnancy may reduce pregnancy‐related complications in PCOS, including preterm birth and pregnancy‐induced hypertension, though its effects on miscarriage and gestational diabetes remain less clear [157, 158]. These findings highlight metformin′s dual potential to improve metabolic and cardiovascular profiles while supporting reproductive outcomes. Thus, metformin represents a cornerstone therapy in PCOS, targeting mitochondrial activity, oxidative stress, and inflammatory signaling to simultaneously improve metabolic, cardiovascular, and reproductive health.

#### 6.2.2. Anti‐Inflammatory Therapies

Pharmacological inhibition of inflammation in PCOS has demonstrated therapeutic potential in both preclinical and clinical models. Although nonsteroidal anti‐inflammatory drugs (NSAIDs) can provide temporary relief of inflammation, their chronic use is discouraged due to associated cardiovascular risks [159]. Targeted suppression of proinflammatory cytokines like TNF‐*α* and IL‐1*β* in PCOS models has resulted in reduced vascular inflammation and oxidative stress. Natural anti‐inflammatory compounds such as curcumin and berberine have been shown to inhibit key inflammatory pathways, particularly NF‐*κ*B and mitogen‐activated protein kinase (MAPK), leading to reduced cytokine expression, lipid peroxidation, and mitochondrial damage [160, 161]. Statins, although primarily lipid‐lowering agents, exhibit anti‐inflammatory effects by reducing hs‐CRP and TNF‐*α* levels while enhancing endothelial function [162]. Some evidence suggests that statins improve mtDNA stability and decrease oxidative stress in PCOS [162]. However, concerns about their potential effects on ovulatory function limit their long‐term use in women of reproductive age.

#### 6.2.3. Antioxidants and Mitochondrial Supportive Agents

Several antioxidant compounds have demonstrated protective roles in PCOS‐related mitochondrial and vascular dysfunction. Molecules such as CoQ10, *N*‐acetylcysteine (NAC), alpha‐lipoic acid, and vitamin E have been observed to lower mitochondrial ROS production, stabilize mitochondrial membrane potential, and preserve mtDNA integrity in experimental models. NAC enhances intracellular antioxidant defenses by promoting GSH synthesis and improves insulin responsiveness [163]. Alpha‐lipoic acid supports mitochondrial ETC activity, particularly at Complexes I and II, thereby promoting vascular dilation and reducing endothelial oxidative stress [164]. Melatonin, known for its mitochondrial‐targeting antioxidant properties, has shown cardioprotective benefits through its ability to stabilize mitochondrial membranes, inhibit the opening of the mitochondrial permeability transition pore (mPTP), and attenuate apoptosis. In PCOS models, melatonin supplementation has been linked to reduced serum CRP levels and improved mitochondrial respiration in cardiac tissue [165]. Although large‐scale clinical trials on antioxidant use in PCOS are limited, preclinical evidence strongly suggests that these agents play a vital role in preserving mitochondrial integrity and mitigating cardiovascular dysfunction in affected individuals.

### 6.3. Novel and Adjunctive Strategies

A growing body of evidence supports the critical role of inflammation in the pathogenesis of CVD, particularly in atherosclerosis and endothelial dysfunction. Therapeutic targeting of proinflammatory cytokines has emerged as a promising adjunctive approach. The Canakinumab Anti‐inflammatory Thrombosis Outcomes Study (CANTOS) offered landmark clinical evidence by demonstrating that canakinumab, a monoclonal antibody against IL‐1*β*, significantly reduced recurrent cardiovascular events in patients with prior myocardial infarction, without altering lipid profiles [118]. This study validated IL‐1*β* as a therapeutic target in atherosclerosis. Other biologics such as anakinra (IL‐1 receptor antagonist) and tocilizumab (IL‐6 receptor antagonist) have shown beneficial effects in small cardiovascular trials, especially among individuals with comorbid inflammatory diseases like rheumatoid arthritis or acute coronary syndrome [118, 166]. However, broader clinical adoption is limited due to concerns about long‐term immunosuppression and infection risk associated with chronic administration. Beyond cytokine blockade, modulation of the NLRP3 inflammasome has garnered attention. The NLRP3 inflammasome links metabolic and mitochondrial dysfunction to the release of IL‐1*β* and IL‐18, key mediators in vascular inflammation. MCC950, a selective NLRP3 inhibitor, has demonstrated efficacy in preclinical models of atherosclerosis and myocardial infarction by attenuating systemic and vascular inflammation [167].

#### 6.3.1. Mitochondria‐Targeted Therapeutics

Given the pivotal role of mitochondrial dysfunction in cardiovascular pathology, via excessive production of mtROS, impaired ATP production, and defective mitophagy, strategies aimed at restoring mitochondrial health are of increasing interest. MitoQ, a mitochondria‐targeted antioxidant composed of CoQ10 linked to a lipophilic triphenylphosphonium cation, selectively accumulates in the mitochondrial matrix. It has shown protective effects in experimental models of endothelial dysfunction, hypertension, and HF by mitigating mitochondrial oxidative stress and preserving vascular integrity [168].

Another promising compound, SS‐31, is a synthetic tetrapeptide that targets the inner mitochondrial membrane. It binds to cardiolipin, a critical phospholipid involved in mitochondrial structure and electron transport, thereby stabilizing mitochondrial membranes, enhancing ATP production, and reducing mtROS. Clinical trials have reported improved mitochondrial bioenergetics in patients with HF, though larger trials are needed to assess its long‐term cardiovascular benefits [169]. Together, these emerging strategies, targeting inflammatory cytokines, inflammasome activation, and mitochondrial dysfunction, represent promising adjuncts to traditional lipid‐lowering and antihypertensive therapies in the prevention and management of CVDs.

## 7. Emerging Role of Short‐Chain Fatty Acids (SCFAs) in PCOS and CVD

SCFAs, mainly acetate, butyrate, and propionate, are key microbial metabolites generated through the fermentation of dietary fibers by the gut microbiota. These SCFAs have been implicated in the regulation of glucose and lipid metabolism, inflammation, oxidative stress, and mitochondrial function, all of which are central to both PCOS and CVD [41].

### 7.1. Butyrate: Mitochondrial and Anti‐Inflammatory Modulator

Butyrate has been extensively studied for its histone deacetylase inhibitory (HDACi) activity, which influences gene transcription involved in inflammation and oxidative stress [170, 171]. Through HDAC inhibition and G‐protein‐coupled receptor (GPR41/43) signaling, butyrate enhances mitochondrial function and ROS and restores metabolic homeostasis.

In PCOS models, butyrate supplementation improves insulin sensitivity, ovarian morphology, and steroidogenic imbalance, alongside a reduction in inflammatory cytokines such as TNF‐*α* and NLRP3 inflammasome activation [172, 173]. Moreover, butyrate positively modulates reproductive function and gut microbiota diversity, an axis often disrupted in PCOS [174, 175]. In cardiovascular models, butyrate improves endothelial function, mitigates oxidative damage, and reduces systemic inflammation, factors crucial in atherosclerosis and HF [176–178]. It also enhances thermogenesis, lipid/glucose metabolism, and appetite regulation [179–182]. Notably, a recent study demonstrated that sodium butyrate administration attenuated cardiorenal and metabolic disturbances in a PCOS model, alongside increasing the antioxidant enzyme paraoxonase‐1 (PON‐1), which is known for hydrolyzing lipid peroxides and protecting LDL/HDL from oxidative modification [23, 183].

#### 7.1.1. Acetate: Abundant, Anti‐Inflammatory, and Cardioprotective

Acetate, the most prevalent SCFA in systemic circulation, exhibits antilipolytic, anti‐inflammatory, antioxidative, and antiapoptotic effects [184, 185]. Like butyrate, it acts through HDAC inhibition and GPR41/43 signaling, influencing key cellular and metabolic pathways [182, 186]. In metabolic and cardiovascular contexts, acetate improves insulin sensitivity, enhances endothelial function, and reduces circulating proinflammatory cytokines. Importantly, recent studies have shown that acetate reverses PCOS‐associated cardiometabolic disturbances, although more work is needed to confirm the mechanism [23, 187, 188].

### 7.2. Implications for Gut–Heart–Ovary Axis in PCOS–CVD Overlap

**Figure 5:**
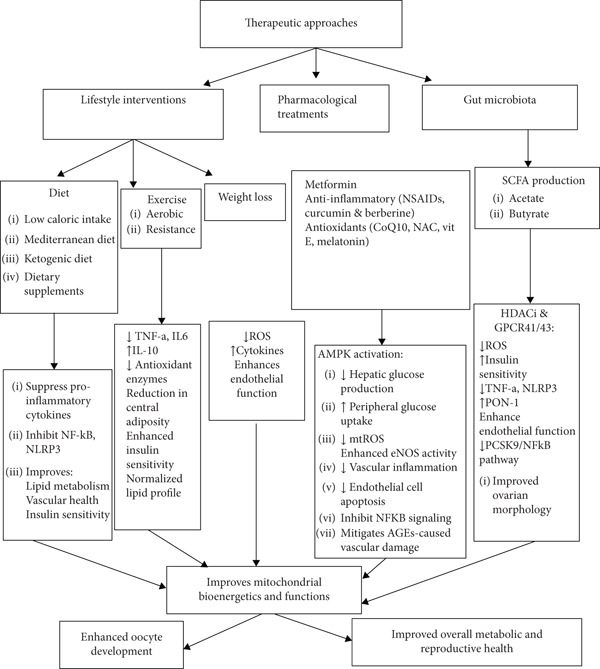
Therapeutic approaches targeting mitochondrial and inflammatory pathways.

Emerging evidence suggests that SCFA deficiency, due to gut dysbiosis, contributes to chronic low‐grade inflammation, oxidative stress, and hormonal imbalances in PCOS, which may predispose individuals to cardiovascular complications [175, 189]. Thus, restoring SCFA levels through dietary interventions or direct supplementation (sodium butyrate or acetate salts) represents a promising adjunct therapy. A consolidated summary of the therapeutic strategies discussed in Sections 6 and 7, including insulin‐sensitizing agents, antioxidant and anti‐inflammatory therapies, mitochondrial‐targeted interventions, hormonal and lifestyle approaches, and emerging SCFA‐based treatments, is presented in Table 1. These interventions, illustrated in Figure 5, collectively target metabolic and reproductive dysfunction by reducing inflammation, improving insulin sensitivity, and enhancing mitochondrial and endothelial function.

**Table 1:** Summary of therapeutic approaches targeting metabolic, inflammatory, and mitochondrial dysregulation in PCOS and CVD, including SCFA‐based strategies.

**Ref**	**Therapeutic approach**	**Examples**	**Primary mechanisms of action**	**Mitochondrial effects**	**Anti-inflammatory/antioxidant effects**	**Cardiovascular/metabolic benefits in PCOS**
[125–131]	Lifestyle interventions	Mediterranean diet, low GI diet, ketogenic diet; aerobic and resistance exercise; weight reduction	Enhances energy balance; improves glucose–lipid metabolism; upregulates PGC‐1*α*; increases NO bioavailability	Improves mitochondrial efficiency; increases biogenesis; reduces ROS production	Suppresses NF‐*κ*B activity; reduces TNF‐*α*/IL‐6; increases IL‐10	Improved endothelial function; reduced arterial stiffness; improved insulin sensitivity; lowered central adiposity
[132, 133]	Dietary supplements	Omega‐3 fatty acids, CoQ10, resveratrol	Modulates lipid metabolism; enhances eNOS and antioxidant enzyme activity	Supports ETC function; stabilizes mtDNA; increases ATP production	Inhibits NF‐*κ*B and NLRP3 inflammasome; reduces oxidative stress	Improved lipid profile; reduced inflammation; enhanced vascular health
[146–150]	Metformin	Standard first‐line PCOS therapy	Activates AMPK; reduces hepatic gluconeogenesis; increases peripheral glucose uptake	Enhances mitochondrial biogenesis and autophagy; reduces mtROS	Inhibits NF‐*κ*B; lowers CRP and TNF‐*α*; reduces AGE‐induced vascular damage	Improved endothelial function, reduced CIMT and arterial stiffness; improved reproductive outcomes and reduced pregnancy complications
[151–155]	Anti‐inflammatory pharmacotherapies	Curcumin, berberine, statins; IL‐1*β* inhibitors; TNF‐*α* blockers	Blocks NF‐*κ*B/MAPK pathways; reduces cytokine secretion	Preserves mitochondrial membrane integrity and reduces mitochondrial damage	Lowers IL‐1*β*, TNF‐*α*, hs‐CRP	Reduced vascular inflammation; improved endothelial function; mitigated oxidative stress
[156–158]	Antioxidants	NAC, alpha‐lipoic acid, vitamin E, melatonin	Enhances glutathione synthesis; improves redox balance	Reduces mtROS; stabilizes mitochondrial membranes; supports ETC function	Reduces lipid peroxidation and oxidative injury	Improved vascular dilation; protection against endothelial dysfunction
[161, 162]	Mitochondria‐targeted therapies	MitoQ, elamipretide (SS‐31)	Directly targets mitochondrial membranes; stabilizes cardiolipin	Decreases mtROS; enhances ATP production; improves mitophagy	Reduces systemic and vascular inflammation	Improved vascular integrity, endothelial homeostasis, and cardiac function
[177–180]	SCFA‐based therapies	Butyrate, acetate	HDAC inhibition; GPR41/43 signaling; metabolic regulation	Enhances mitochondrial respiration; reduces ROS	Decreases TNF‐*α*, IL‐1*β*, NLRP3 activity; improves antioxidant responses	Improved endothelial function; reduced oxidative stress; improved insulin sensitivity and cardiometabolic profiles

## 8. Future Research Directions

Advancing the understanding of cardiovascular risk in PCOS requires more phenotype‐specific investigations, as the metabolic and cardiovascular burden differs substantially between insulin‐resistant, obese, hyperandrogenic, and normoandrogenic subtypes. Future randomized controlled trials should therefore be designed to stratify participants by PCOS phenotype to determine whether mitochondria‐targeted agents, such as CoQ10, MitoQ, or SS‐31, and anti‐inflammatory therapies, including TNF‐*α* inhibitors or IL‐1 receptor antagonists, exert differential cardiometabolic benefits across these subgroups.

Given the multifactorial nature of PCOS, exploring combination therapeutic strategies represents another important direction. Integrating lifestyle modification with pharmacologic interventions such as metformin, resveratrol, or antioxidant therapy may produce synergistic improvements in mitochondrial biogenesis, systemic inflammation, and vascular function. Similarly, adjunctive therapies that restore gut‐derived metabolites such as SCFAs could augment the effects of conventional treatments by targeting the gut–heart–ovary axis. Additionally, mechanistic studies should further investigate the bidirectional interactions between mitochondrial injury and chronic inflammation, particularly how early disturbances in these pathways contribute to long‐term cardiovascular remodeling. The development of robust and accessible biomarkers reflecting mitochondrial efficiency, oxidative stress, NLRP3 activity, or inflammatory load would greatly enhance both risk stratification and therapeutic monitoring.

Finally, integrating genomic, metabolomic, and microbiome‐based profiling into clinical research may enable the development of precision medicine frameworks, allowing treatment selection to be tailored to an individual′s molecular phenotype. Such multidimensional approaches, combining endocrinology, cardiovascular science, reproductive medicine, and systems biology, are essential for translating emerging mechanistic insights into targeted interventions capable of reducing the lifelong cardiometabolic burden in women with PCOS.

## 9. Conclusion

Cardiovascular dysfunction is a significant, yet often underrecognized, complication of PCOS. Emerging evidence highlights the interconnected roles of mitochondrial dysfunction and chronic low‐grade inflammation in driving this risk. Mitochondrial abnormalities impair energy metabolism, elevate ROS, and activate inflammatory pathways that contribute to endothelial injury, IR, and atherogenesis. Simultaneously, systemic inflammation, amplified by adipose tissue dysfunction and hormonal imbalance, reinforces mitochondrial defects, creating a self‐perpetuating loop that accelerates cardiovascular deterioration. As a result, women with PCOS face an elevated lifetime risk of hypertension, dyslipidemia, IR, metabolic syndrome, and other cardiometabolic disturbances. Understanding these interconnected molecular mechanisms provides a strong foundation for developing more targeted therapeutic strategies. Interventions that restore mitochondrial integrity, reduce oxidative stress, and modulate inflammatory signaling may offer substantial benefit in mitigating cardiovascular risk in the PCOS population. Looking forward, deeper integration of mechanistic research with clinical practice will be essential. Precision phenotyping and well‐designed intervention studies, particularly those evaluating combination therapies, hold promise for improving cardiovascular outcomes and overall quality of life for women living with PCOS.

**Nomenclature**
CIMT: carotid intima–media thickness
HIF‐1*α*
: hypoxia‐inducible factor 1‐alpha
MFN2: mitofusin‐2
AMH: anti‐Müllerian hormone
ACTH: adrenocorticotropic hormone
SHBG: sex hormone–binding globulin
HNF‐4*α*
: hepatocyte nuclear factor 4‐alpha
IGFBP‐1: insulin‐like growth factor–binding Protein 1
IGF‐1: insulin‐like growth factor‐1
AFC: antral follicle count
ApoB‐containing VLDL: Apolipoprotein B–containing very low‐density lipoprotein (VLDL)
sLOX‐1: soluble lectin–like oxidized low‐density lipoprotein receptor‐1
mtDNA: mitochondrial DNA
ETC: electron transport chain
NOX: nicotinamide adenine dinucleotide phosphate oxidase
MnSOD: manganese superoxide dismutase
DAMPs: damage‐associated molecular patterns
hs‐CRP: high‐sensitivity C‐reactive protein
NETs: neutrophil extracellular traps
Th1: T Helper 1
Th17: T Helper 17
MMPs: matrix metalloproteinases
TLRs: Toll‐like receptors
NLRP3: NOD‐like receptor Protein 3 inflammasome
OXPHOS: oxidative phosphorylation
IFN‐*γ*
: interferon‐gamma
JAK/STAT: Janus kinase/signal transducer and activator of transcription
TGF‐*β*
: transforming growth factor‐beta
PI3K/AKT: phosphoinositide 3‐kinase/protein kinase B pathway
NF‐*κ*B: nuclear factor kappa‐light‐chain‐enhancer of activated B cells
PRRs: pattern recognition receptors
cGAS–STING pathway: cyclic GMP‐AMP synthase–stimulator of interferon genes pathway
PGC‐1*α*
: peroxisome proliferator–activated receptor gamma Coactivator 1‐alpha
MitoQ: mitochondria‐targeted antioxidant derived from coenzyme Q10
SS‐31: elamipretide
CoQ10: coenzyme Q10
VO_2_ max: maximal oxygen uptake
AMPK: AMP‐activated protein kinase
MAPK: mitogen‐activated protein kinase
NAC: *N*‐acetylcysteine
mPTP: mitochondrial permeability transition pore
CANTOS: Canakinumab Anti‐inflammatory Thrombosis Outcomes Study
MCC950: a selective NLRP3 inflammasome inhibitor
mtROS: mitochondrial reactive oxygen species
PON‐1: paraoxonase‐1 HDAC
GPR41/43: G‐protein‐coupled Receptors 41 and 43
PCSK9: proprotein convertase subtilisin/kexin Type 9

## Ethics Statement

The authors have nothing to report.

## Consent

The authors have nothing to report.

## Disclosure

All authors reviewed and approved the final version of the manuscript for submission.

## Conflicts of Interest

The authors declare no conflicts of interest.

## Author Contributions

O.C.B., M.O.A., O.E.C‐A., M.V.O., S.O.O., O.A.S., O.A.B., and T.O.O. drafted the manuscript and performed data extraction and curation. O.C.B. and M.O.A. edited and revised the manuscript.

## Funding

No funding was received for this manuscript.

## Data Availability

The authors have nothing to report.
